# The Role of Heterogenous Real-world Data for Dengue Surveillance in Martinique: Observational Retrospective Study

**DOI:** 10.2196/37122

**Published:** 2022-12-22

**Authors:** Emmanuelle Sylvestre, Elsa Cécilia-Joseph, Guillaume Bouzillé, Fatiha Najioullah, Manuel Etienne, Fabrice Malouines, Jacques Rosine, Sandrine Julié, André Cabié, Marc Cuggia

**Affiliations:** 1 Laboratoire de Traitement du Signal et de l'Image (LTSI) - Unité Mixte de Recherche (UMR) 1099 Université de Rennes, Centre Hospitalier Universitaire Rennes Institut national de la santé et de la recherche médicale (INSERM) Rennes France; 2 Centre de Données Cliniques Centre Hospitalier Universitaire Martinique Fort-de-France Martinique; 3 Laboratoire de Virologie Centre Hospitalier Universitaire Martinique Fort-de-France Martinique; 4 Centre de Démoustication et de Recherche Entomologique Collectivité Territoriale de la Martinique – Agence Régionale de Santé Fort-de-France Martinique; 5 Cellule Martinique Santé Publique France Saint-Maurice France; 6 Département d'Information Médicale Service de Santé Publique Centre Hospitalier Universitaire Martinique Fort-de-France Martinique; 7 Infectious and Tropical Diseases Unit Centre Hospitalier Universitaire Martinique Fort-de-France Martinique; 8 Centre d'Investigation Clinique (CIC)-1424 Centre Hospitalier Universitaire Martinique Institut national de la santé et de la recherche médicale (INSERM) Fort-de-France Martinique; 9 Pathogenesis and Control of Chronic and Emerging Infections (PCCEI) Université de Montpellier - Université des Antilles Institut national de la santé et de la recherche médicale (INSERM) - Etablissement Français du Sang (EFS) Montpellier France

**Keywords:** dengue, surveillance, real-word data, Big Data, Caribbean, dengue-endemic region

## Abstract

**Background:**

Traditionally, dengue prevention and control rely on vector control programs and reporting of symptomatic cases to a central health agency. However, case reporting is often delayed, and the true burden of dengue disease is often underestimated. Moreover, some countries do not have routine control measures for vector control. Therefore, researchers are constantly assessing novel data sources to improve traditional surveillance systems. These studies are mostly carried out in big territories and rarely in smaller endemic regions, such as Martinique and the Lesser Antilles.

**Objective:**

The aim of this study was to determine whether heterogeneous real-world data sources could help reduce reporting delays and improve dengue monitoring in Martinique island, a small endemic region.

**Methods:**

Heterogenous data sources (hospitalization data, entomological data, and Google Trends) and dengue surveillance reports for the last 14 years (January 2007 to February 2021) were analyzed to identify associations with dengue outbreaks and their time lags.

**Results:**

The dengue hospitalization rate was the variable most strongly correlated with the increase in dengue positivity rate by real-time reverse transcription polymerase chain reaction (Pearson correlation coefficient=0.70) with a time lag of −3 weeks. Weekly entomological interventions were also correlated with the increase in dengue positivity rate by real-time reverse transcription polymerase chain reaction (Pearson correlation coefficient=0.59) with a time lag of −2 weeks. The most correlated query from Google Trends was the “Dengue” topic restricted to the Martinique region (Pearson correlation coefficient=0.637) with a time lag of −3 weeks.

**Conclusions:**

Real-word data are valuable data sources for dengue surveillance in smaller territories. Many of these sources precede the increase in dengue cases by several weeks, and therefore can help to improve the ability of traditional surveillance systems to provide an early response in dengue outbreaks. All these sources should be better integrated to improve the early response to dengue outbreaks and vector-borne diseases in smaller endemic territories.

## Introduction

Dengue is one of the most important vector-borne diseases worldwide, with 390 million infections, 96 million symptomatic cases, and 20,000 estimated deaths per year in >125 countries [1,2]. The disease is mostly endemic in tropical and subtropical regions (ie, Southeast Asia, the Americas, and the Pacific), with 4 billion people at risk [3]. In Latin America and the Caribbean, morbidity and mortality increased from 400,519 cases and 92 deaths in 2000 to >3.1 million cases and 1534 deaths in 2019 [4,5]. Dengue prevention and control in these regions rely on 2 main approaches: vector control programs and traditional surveillance, which is based on passive detection of symptomatic cases (inpatients and outpatients) [4,6]. Although both approaches are effective, they are expensive and are hampered by the delay between case occurrence and case reporting. Furthermore, some countries do not have routine vector control measures [7] and national epidemiological surveillance systems tend to underestimate the true disease burden of dengue [8].

In Martinique, a French overseas territory in the Lesser Antilles with approximately 360,000 inhabitants, health authorities have launched the “Monitoring, warning and management of dengue outbreaks program” (Programme de surveillance, d’alerte et de gestion des épidémies de dengue [PSAGE]), in which vector control and traditional surveillance are combined. PSAGE identifies five main stages in dengue outbreaks: (1) sporadic transmission, (2) dengue clusters with or without an epidemiological link, (3) epidemic risk when the number of symptomatic cases is above the expected threshold, (4) dengue outbreak, and (5) return to normal. Vector surveillance still plays a role in this system; however, the change in PSAGE stage is mainly based on the number of symptomatic cases identified by general practitioners who are part of the French Sentinel Network surveillance system [9,10].

Surveillance systems are a key public health tool to detect early cases of emerging infectious diseases, prevent outbreaks [11] among populations, and implement measures to reduce transmission [12]. Traditional surveillance systems are often expensive because of the time and resources required to process data collected from public health networks [13]. To improve these systems and reduce the delay between diagnosis and reporting, researchers have evaluated novel data sources, especially real-world data (ie, data not collected in experimental conditions [14]), such as emergency department visits, mobile data, and internet-based systems [15-18]. Other studies on surveillance and forecasting, especially those using climate data [19-21], have also shown promising results. Scientists mostly rely on correlation methods to test these data sources [22,23], but other approaches have also been tested, for instance Naive Bayes methods [24,25]. Most of these studies were conducted in Asia (70% of the global dengue burden) [2]. Studies in the Americas concerned large territories or countries, such as Brazil and Mexico [24,25], and in the Caribbean, they focused on the bigger islands of the Greater Antilles [21,26].

The aim of this study, carried out in Martinique, was to investigate whether heterogeneous real-world data sources could help to reduce reporting delays and improve dengue monitoring in a smaller endemic region.

## Methods

### Data Sources

#### Overview

We used several types of data that had been routinely collected during the study period (from January 1, 2007, to February 28, 2021): epidemiological surveillance reports from the French National Public Health Agency (Santé Publique France), reimbursement claims and laboratory data from Martinique University Hospital, entomological data from the Martinique Mosquito Control and Entomological Research Center (Centre de Démoustication et de Recherche Entomologique [CEDRE]), and relative search volumes (RSV) from Google Trends. Entomological, clinical, and laboratory data are available within 24 to 48 hours. Google RSV and epidemiological surveillance data are available in real time and at the end of each week, respectively. All used data were anonymized.

#### Epidemiological Surveillance Data

We obtained weekly dengue surveillance reports from the French Public Health Agency. These reports are based on data collected by general practitioners from the French Sentinel Network. They also provide the official start and end dates of each dengue outbreak and the weekly PSAGE stage during the outbreak. These reports are not continuously published but only if the dengue risk level is above stage 1 (ie, the baseline stage). We used the PSAGE stage described in each report to create the PSAGE ordinal variable with 4 levels. Indeed, although the PSAGE program has 5 levels, stage 5 (“back to normal”) was used only 5 times in the last 15 years, and experts prefer to use stage 1 (“sporadic transmission”) after stage 4 (“dengue outbreak”). Moreover, when stage 5 was used, it was for 1 week, except once in 2021, when it lasted 2 weeks. Thus, we combined stages 1 and 5 into a single stage (stage 1 or 5, sporadic transmission).

#### Clinical and Laboratory Data

We obtained weekly aggregated data from Martinique University Hospital: (1) inpatient data (age and diagnoses associated with dengue disease or dengue symptoms), (2) administrative data (outpatient medical consultations, hospitalizations, and emergency department visits), and (3) laboratory data—dengue virus (DENV) detection by real-time reverse transcriptase polymerase chain reaction (RT-PCR).

All included diagnoses were coded using the French version of the International Classification of Diseases, 10th edition (ICD-10):

- Dengue or severe dengue
- Possible coding errors associated with dengue: fever and unspecified viral hemorrhagic fever
- Severity symptoms: hemorrhage, shock, and dehydration
- Thrombocytopenia
- Hepatic symptoms: hepatitis, hepatomegaly, hepatic failure, and elevation of transaminase
- Neurological symptoms: encephalitis and encephalopathy

We selected these diagnoses with the help of infectious disease physicians. All reimbursement claims data were obtained from the Martinique University Hospital, where the only infectious disease department for the whole island is located. The relevant ICD-10 codes are listed in Multimedia Appendix 1.

We normalized all administrative data as follows:

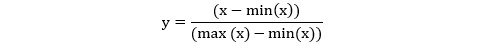



where *x* is the weekly number of hospitalizations, consultations, or emergency department visits and min and are the minimum and maximum values observed in the data set, respectively.

For laboratory data, we used the DENV positivity rate determined by RT-PCR. Laboratory results were concerned about both inpatients and outpatients because the Martinique University Hospital is the reference center for DENV screening using RT-PCR in Martinique.

#### Entomological Data

We used data from the CEDRE surveillance database, such as the weekly number of entomological interventions and where they were carried out. Entomological interventions were defined as all vector control interventions and measures taken by CEDRE: information and education of the households, physical vector control (ie, eliminating mosquito sites, such as old containers filled with water), and chemical vector control with insecticides [26]. This agency manages entomological surveillance and vector control in Martinique and collects data on each intervention.

#### Google RSV

We used data from Google Trends [27], which provides real-time and archived information on Google queries from 2004 onward. These queries are normalized by Google as RSV by dividing the total search volume for a query in a geographic location by the total number of queries in that region at a given point in time [28]. We used this tool to retrieve information on the search interest for keywords associated with dengue during our selected time frame (January 2007 to February 2021). However, we could not retrieve weekly data for the Martinique region, especially for the first years of the study period, because there were not always enough RSV (as indicated by the Google error message “Sorry, not enough search volume to show graphs”). Therefore, we based our methodology to retrieve Google Trends data on previously published methodology frameworks, indicating that Google Trends data should be retrieved for exactly the same period as the other data under study and as a single data set rather than as individual queries for each year [29]. As data for our study period were only available at monthly intervals, we considered that interest was constant over each week of the month for each query.

For data retrieval, relevant keywords were selected with experts in the field. Normally, all spelling variations should be included in the research to limit the risk of missing data. However, in our case, combining all possible spelling variants of some keywords into a single query was impossible, and an error message from Google indicated that the available data were insufficient. Nevertheless, we retrieved results using the “topic” option from Google that includes various keywords associated with a category.

As Martinique (and the other islands in the Lesser Antilles) are small regions, we tried 2 strategies to explore the geographic region of our keywords: we selected “Martinique” as the region in the tool and we added “Martinique” as a keyword in our query, with the region selected as “worldwide.” Moreover, we selected our keywords in 3 different languages (French, English, and Spanish) because the Lesser Antilles is a multilingual region.

### Data Processing

Clinical and laboratory data were already aggregated into a structured database and did not require data processing. Similarly, data from Google queries are normalized by Google as RSV. Conversely, most of the information in the CEDRE database was unstructured and required processing. Indeed, the CEDRE database is a comprehensive database with some structured data (eg, the date of an entomological intervention), but the details associated with entomological interventions (ie, the type of insecticide used or the number of old containers removed) were in free text; we needed this information to count the number of weekly interventions. Therefore, we used rule-based natural language processing methods (ie, part-of-speech tags) to process the data and extract relevant information for our study. All statistical analyses were performed using R (version 4.1.0; R Foundation for Statistical Computing) [30] (*tidytext* [31]*,* *stopwords*, and *SnowballC* packages).

### Statistical Analysis

A total of 4 dengue outbreaks were recorded in Martinique between 2007 and 2021: from August 20, 2007, to January 14, 2008; from February 22, 2010, to October 25, 2010; from July 22, 2013, to April 14, 2014; and from November 18, 2019, to February 8, 2021. The fourth dengue outbreak was the largest in Martinique over the last 20 years.

During the same period, there was a chikungunya outbreak in 2014, a Zika virus outbreak in 2016, the first COVID-19 wave in March 2020, and the second COVID-19 wave from September to December 2020. The last dengue outbreak was concomitant with the second COVID-19 wave. Consequently, the PSAGE stages did not vary much over the years, making it difficult to study correlations with this categorical variable. Therefore, it is necessary to find a good continuous estimator for time series analyses. To this end, we assessed the DENV RT-PCR positive rate performance for PSAGE stage prediction using a repeated stratified k-fold cross-validation approach. First, we divided the data into 10 stratified folds, then built a logistic regression model to predict the PSAGE stages. Finally, we repeated the process 10 times and evaluated its performance. The original PSAGE variable was an ordinal variable with 4 levels, but the data were not evenly distributed among the levels. Therefore, we ran 4 binary logistic regression analyses, rather than a single multinomial regression model, to assess how RT-PCR positive rates can predict each level. We calculated the predicted probability of a PSAGE stage by using the following equation:

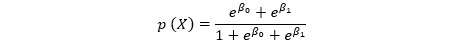



where X is the vector of the predictor values, β_1_ is the vector of the regression coefficients, and β_0_ is the intercept of the model. As the data set was imbalanced, we also used stratified sampling in the PSAGE stage for k-fold cross-validation.

The metrics used to assess the logistic model performance were accuracy, specificity, precision, recall, *F_1_*-score, and area under the curve (AUC).

Accuracy assesses the overall effectiveness of the logistic regression model and can be defined as the ratio of the correct number of predictions to the total number of predictions:

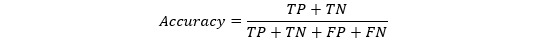



where TP are true positive, TN are true negative, FP are false positive, and FN are false negative results.

Specificity is the model’s capacity to predict that a week is not in the PSAGE stage and is defined as the ratio between correctly predicted negative classes and all items that are actually negative:

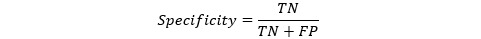



where TN is true negative, and FP is false positive.

Precision (or positive predictive value) is the agreement between the true stages and the stages predicted by the RT-PCR positive rate and is defined as the ratio between the correctly predicted positive classes and all items predicted to be positive:

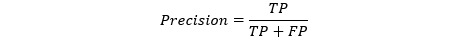



where TP is true positive, and FP is false positive.

Recall (or sensitivity) is the model’s capacity to identify the true stages and is defined as the ratio between correctly predicted positive classes and all items that are actually positive:

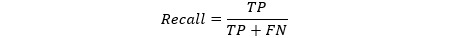



where TP is true positive, and FN is false negative.

The *F*_1_-score is the harmonic mean of precision and recall. The AUC represents the capacity of the model to avoid false classification into a stage.

To investigate the association between the RT-PCR positive rate and each data source, we plotted their time series. Finally, for each source, we estimated the Pearson correlation coefficient (*r*) and the cross-correlations between the weekly data and the DENV RT-PCR positive rate. The aim of the cross-correlation function is to investigate the relationship between time series and their lag values [32]. In our case, we wanted to determine whether the increase in the studied variables was correlated with the DENV RT-PCR positive rate and whether it preceded it. All statistical analyses were performed using R (version 4.1.0) [30]. For cross-correlations, significance is determined graphically when the lines are above (or below) the dotted blue line.

### Ethics Approval

This study was approved by the local Ethics Committee of Martinique University Hospital (approval number 2022/177).

## Results

### RT-PCR Positive Rate Performance

The accuracy and AUC values ranged between 0.83 and 0.95 and between 0.55 and 0.89, respectively. Overall, the model performed better at predicting sporadic transmission (stage 1 or 5: accuracy=0.83; AUC=0.84) and outbreak (stage 4: accuracy=0.89; AUC=0.89; Table 1).

**Table 1:** DENV^a^ RT-PCR^b^ positive rate and PSAGE^c^ stage prediction.

Metrics	PSAGE			
	Stage 1 or 5	Stage 2	Stage 3	Stage 4
Accuracy	0.828	0.879	0.953	0.888
Specificity	0.616	1	1	0.96
Precision	0.827	—^d^	—	0.742
Recall	0.936	0	0	0.535
*F*_1_-score	0.878	—	—	0.612
AUC^e^	0.838	0.546	0.828	0.888

^a^DENV: dengue virus.

^b^RT-PCR: real-time reverse transcriptase polymerase chain reaction.

^c^PSAGE: Programme de surveillance, d’alerte et de gestion des épidémies de dengue.

^d^Not enough data available to build a prediction model for these stages.

^e^AUC: area under the curve.

### Hospital Data

We normalized all hospital data to plot the time series to consider the different scales. As children and adults can be affected differently depending on the dengue infection type (primary vs secondary), we stratified our data sets based on the ward type (adult or pediatric).

#### Administrative Data

Adult hospitalizations (*P*=.01) and emergency department visits (*P*<.001) were significantly correlated with the DENV RT-PCR positive rate. We also observed a significant cross-correlation at −3 and −5 weeks, suggesting that the increase in emergency department visits preceded the increase in the DENV RT-PCR positive rate by 3 to 5 weeks. Table 2 shows the correlations and cross-correlations between the administrative data and DENV detection rate by RT-PCR. All cross-correlations between administrative data and DENV RT-PCR positive rate are listed in Multimedia Appendix 2.

**Table 2:** Correlations and cross-correlations between administrative data and DENV^a^ RT-PCR^b^ positive rate.

Data		Correlation (95% CI)	*P* value	Max cross-correlation^c^	Time lag^d^
**Hospitalizations**					
	Total (n=506,992)	−0.066 (−0.137 to 0.006)	.07	−0.091	−5 weeks
	Adults (n=444,045)	−0.095 (−0.165 to −0.023)	*.01* ^e^	−0.097	−4 weeks
	Children (n=62,947)	0.067 (−0.004 to 0.139)	.06	*0.118* ^f^	−*8 weeks*
**Emergency department visits**					
	Total (n=1,082,343)	0.111 (0.039 to 0.181)	*.002*	*0.169*	−*5 weeks*
	Adults (n=740,282)	0.181 (0.11 to 0.25)	*<.001*	0.216	−3 weeks
	Children (n=342,061)	0.046 (−0.025 to 0.118)	.21	*0.107*	−*5 weeks*
**Consultations**					
	Total (n=2,715,906)	−0.065 (−0.137 to 0.007)	.08	−0.067	−2 weeks
	Adults (n=2,467,565)	−0.061 (−0.133 to 0.0105)	.09	−0.097	−5 weeks
	Children (n=248,341)	−0.046 (−0.118 to 0.026)	.21	−0.087	−5 weeks

^a^DENV: dengue virus.

^b^RT-PCR: real-time reverse transcriptase polymerase chain reaction.

^c^Maximum cross-correlation.

^d^Time lag that results in the maximum cross-correlation.

^e^Italicized *P* values are significant.

^f^Italicized cross-correlations are statistically significant (details in Multimedia Appendices 2-5).

#### Inpatient Data

We normalized inpatient data as the percentage of each diagnosis among all diagnoses for that year. The percentage of dengue diagnoses among inpatients was significantly associated with an increase in the DENV RT-PCR positive rate. We also detected a significant cross-correlation at −3 weeks, indicating that the increase in dengue diagnoses among hospitalized people preceded the increase in DENV RT-PCR positive rates by 3 weeks (Table 3). All cross-correlations between dengue diagnoses in inpatients and DENV RT-PCR positive rates are listed in Multimedia Appendix 3.

Concerning dengue-related symptoms, thrombocytopenia and liver involvement in adults and children were associated with the DENV RT-PCR positive rate.

The significant cross-correlation, at time lags ranging between −2 and −5 weeks, indicated that the increase in thrombocytopenia and liver dysfunction preceded the increase in DENV RT-PCR positive rates by 3 to 5 weeks (Table 4). All cross-correlations between dengue symptoms among inpatients and DENV RT-PCR positive rate are listed in Multimedia Appendices 4 and 5.

**Figure 1:**
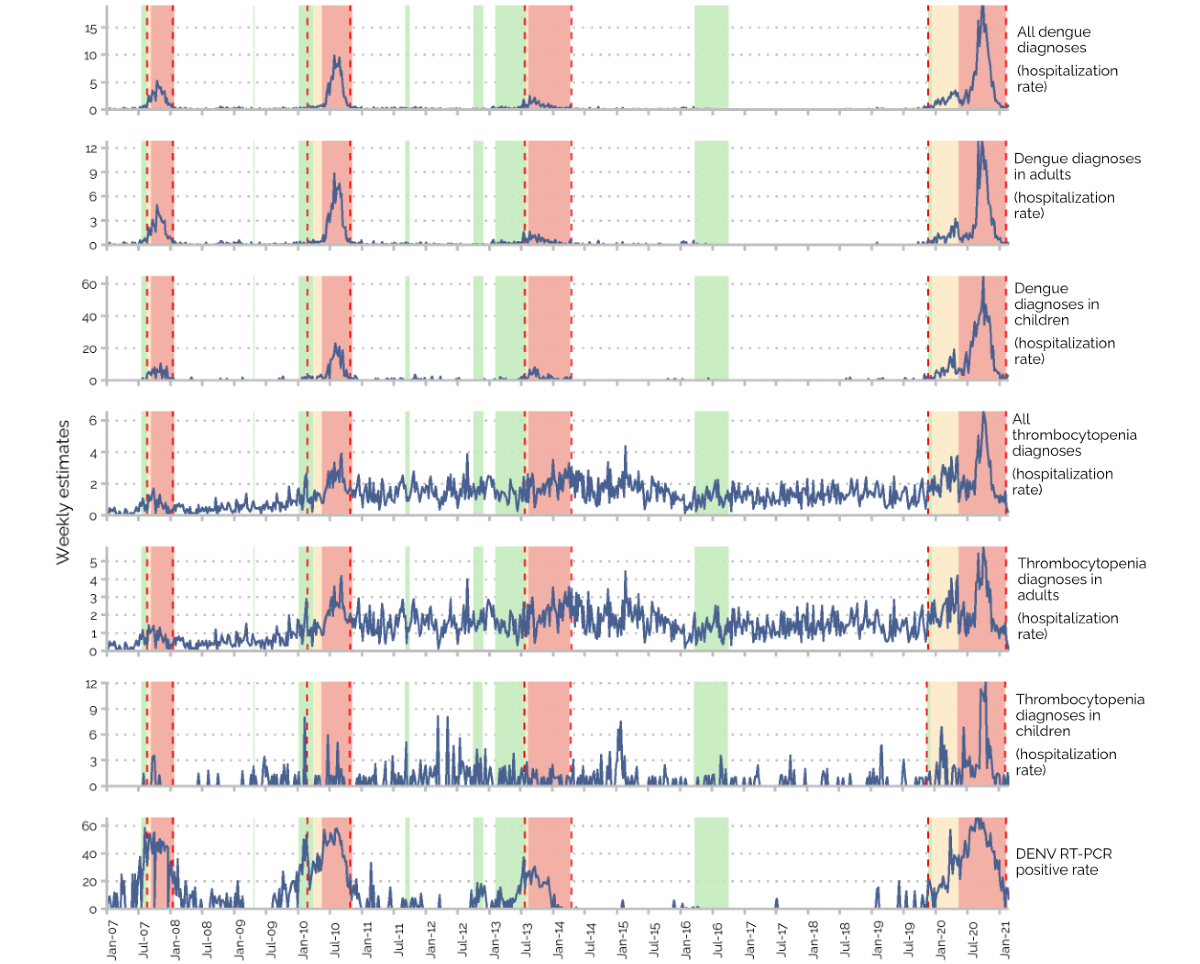
Weekly hospitalization rates for dengue and thrombocytopenia during the different Programme de surveillance, d’alerte et de gestion des épidémies de dengue (PSAGE) stages from January 2007 to February 2021. The DENV RT-PCR positive rate was used as a reference. Blue curves: weekly hospitalization rates for the indicated ICD-10 diagnoses. Green areas: PSAGE stage 2 (dengue clusters). Yellow areas: PSAGE stage 3 (epidemic risk). Red areas: PSAGE stage 4 (dengue outbreak). Red dashed lines: official dates of dengue outbreaks that were decided retrospectively by the French Public Health Agency at the end of each outbreak. DENV: dengue virus; RT-PCR: real-time reverse transcription polymerase chain reaction.

The weekly hospitalization rates for dengue and thrombocytopenia during the study period are shown in Figure 1, with DENV RT-PCR positive rate, as a reference.

**Table 3:** Correlations between dengue diagnoses inpatients and DENV^a^ RT-PCR^b^ positive rate.

Data	Correlation (95% CI)	*P* value	Max cross-correlation^c^	Time lag^d^
Total	0.704 (0.665-0.738)	*<.001* ^e^	*0.710* ^f^	−*3 weeks*
Adults	0.698 (0.659-0.733)	*<.001*	*0.703*	−*3 weeks*
Children	0.672 (0.631-0.701)	*<.001*	*0.675*	−*3 weeks*

^a^DENV: dengue virus.

^b^RT-PCR: real-time reverse transcriptase polymerase chain reaction.

^c^Maximum cross-correlation.

^d^Time lag that results in the maximum cross-correlation.

^e^Italicized *P* values are significant.

^f^Italicized cross-correlations are statistically significant (details in Multimedia Appendices 2-5).

**Table 4:** Correlations between dengue symptoms among inpatients and dengue RT-PCR^a^ positive rate.

Data		Correlation (95% CI)	*P* value	Max cross-correlation^b^	Time lag^c^
**Symptoms**					
	Total	0.077 (0.005 to 0.148)	*.04* ^d^	*0.081* ^e^	−*4 weeks*
	Adults	0.071 (−0.001 to 0.142)	.05	0.071	0 weeks
	Children	0.093 (0.021 to 0.16)	*.01*	*0.127*	−*4 weeks*
**Coding errors**					
	Total	−0.096 (−0.167 to –0.024)	*.009*	−0.098	−1 week
	Adults	−0.072 (−0.143 to –1.12 ×10^−4^)	*.05*	−0.086	−2 weeks
	Children	−0.043 (−0.115 to 0.0285)	.24	−0.043	0 weeks
**Symptom severity**					
	Total	0.105 (0.033 to 0.175)	*.004*	0.105	0 weeks
	Adults	0.068 (−0.004 to 0.139)	.07	0.068	0 weeks
	Children	0.263 (0.195 to 0.329)	<.001	0.279	−4 weeks
**Thrombocytopenia**					
	Total	0.281 (0.213 to 0.346)	*<.001*	*0.289*	−*2 weeks*
	Adults	0.235 (0.166 to 0.302)	*<.001*	*0.242*	−*2 weeks*
	Children	0.269 (0.201 to 0.335)	*<.001*	*0.288*	−*4 weeks*
**Liver dysfunction symptoms**					
	Total	0.152 (0.081 to 0.222)	*<.001*	*0.179*	−*5 weeks*
	Adults	0.123 (0.0517 to 0.193)	*<.001*	*0.153*	−*5 weeks*
	Children	0.152 (0.081 to 0.222)	*<.001*	*0.147*	−*5 weeks*
**Neurological symptoms**					
	Total	−0.028 (−0.100 to 0.0435)	.44	−0.061	−7 weeks
	Adults	−0.045 (−0.117 to 0.0265)	.22	−0.068	−7 weeks
	Children	0.029 (−0.0427 to 0.101)	.43	0.034	−6 weeks

^a^RT-PCR: real-time reverse transcriptase polymerase chain reaction.

^b^Maximum cross-correlation.

^c^Time lag that results in the maximum cross-correlation.

^d^Italicized *P* values are significant.

^e^Italicized cross-correlations are statistically significant (details in Multimedia Appendices 2-5).

### Entomological Data

The weekly number of entomological interventions was significantly (*P*<.001) associated with DENV RT-PCR positive rate (*r*=0.591; 95% CI 0.542-0.636). They were also significantly cross-correlated (0.627 at −2 weeks), indicating that their increase preceded an increase in the DENV RT-PCR positive rate by 2 weeks.

We did not find any significant correlation or cross-correlation between the intervention zones and the RT-PCR positive rate.

### Google RSV

We considered that interest was constant over each week of the month for each query to compute our weekly data, but RSV could have high variability across weeks. Therefore, we also compared monthly RSV to monthly DENV RT-PCR positive rates to assess whether our approach had a high impact on the results.

Several Google keywords were significantly associated with the DENV RT-PCR positive rate. Overall, this association was stronger for the simplest combination of keywords, without spelling variations, especially for the keywords “dengue symptoms.” We could not assess some keyword combinations because of the lack of data. Furthermore, when Google Trends provided “Topics,” the results outperformed those obtained using manual combinations of keywords that included spelling, language, or accent variations. Keywords not restricted to the geographic region of “Martinique” (by using the Geographical region feature or by adding the keyword “Martinique” to the query) were not significantly associated with the DENV RT-PCR positive rate. We obtained the strongest significant cross-correlation using the topic “dengue” in the Martinique region (0.643 at the time lag of −3 weeks). This indicated that an increase in queries for this term in the Martinique region preceded the increase in the DENV RT-PCR positive rate by 3 weeks (Table 5). Conversely, we did not find any significant cross-correlation within meaningful time lag values for the term “mosquito” and its different spellings and language variations.

For monthly correlations, the results were similar to weekly results (Table 6). All weekly correlations between Google Trends keywords and DENV RT-PCR positive rates are listed in Multimedia Appendix 6. All monthly correlations between Google Trends keywords and DENV RT-PCR positive rates are listed in Multimedia Appendix 7. All weekly cross-correlations between nonhospital data and DENV RT-PCR positive rate are listed in Multimedia Appendix 8. All monthly cross-correlations between Google Trends keywords and DENV RT-PCR positive rate are listed in Multimedia Appendix 9.

**Figure 2:**
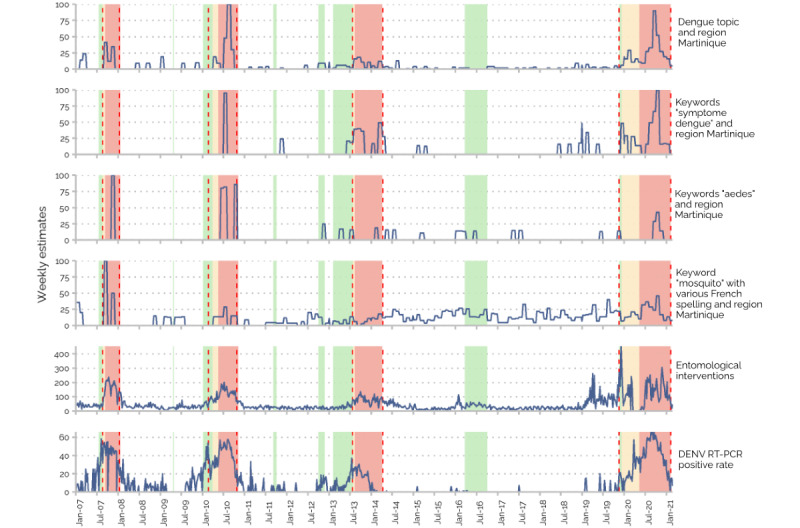
Weekly estimates for the indicated nonhospital data during the different Programme de surveillance, d’alerte et de gestion des épidémies de dengue (PSAGE) stages from January 2007 to February 2021. The DENV RT-PCR positive rate was used as a reference. Blue curves: weekly estimates for the strongest correlated Google keywords and entomological interventions. Green areas: PSAGE stage 2 (dengue clusters). Yellow areas: PSAGE stage 3 (epidemic risk). Red areas: PSAGE stage 4 (dengue outbreak). Red dashed lines: official dates of the outbreaks decided retrospectively by the French Public Health Agency at the end of each outbreak. DENV: dengue virus; RT-PCR: real-time reverse transcription polymerase chain reaction.

The weekly estimates for nonhospital data during the study period are displayed in Figure 2, with the DENV RT-PCR positive rate as a reference.

**Table 5:** Strongest correlations between Google Trends keywords and DENV^a^ RT-PCR^b^ positive rate.

Keywords			Correlation (95% CI)		*P* value		Max cross-correlation^c^		Time lag^d^
**Dengue**									
	Keywords “dengue” + “dingue” and region “Martinique”	0.597 (0.548-0.641)		*<.001* ^e^		*0.598* ^f^		−*1 week*	
	Keywords “dengue” + “Martinique”	0.534 (0.480-0.583)		*<.001*		*0.611*		−*6 weeks*	
	Topic “dengue” and region “Martinique”	0.637 (0.591-0.677)		*<.001*		*0.643*		−*3 weeks*	
**Dengue symptoms**									
	Keyword “symptome dengue” and region “Martinique”	0.412 (0.351-0.47)		*<.001*		*0.435*		−*3 weeks*	
**Mosquito**									
	Keyword “mosquito” with various French spellings and region “Martinique”	0.200 (0.130-0.268)		*<.001*		0.200		0 weeks	
**Aedes**									
	Keywords “aedes” and region “Martinique”	0.339 (0.273-0.401)		*<.001*		*0.369*		−*3 weeks*	
	Topic “aedes” and region “Martinique”	0.214 (0.591-0.677)		*<.001*		*0.304*		−*7 weeks*	

^a^DENV: dengue virus.

^b^RT-PCR: real-time reverse transcriptase polymerase chain reaction.

^c^Maximum cross-correlation.

^d^Time lag that results in the maximum cross-correlation.

^e^Italicized *P* values are significant.

^f^Italicized cross-correlations are statistically significant (details in Multimedia Appendices 2-5).

**Table 6:** Strongest monthly correlations between Google Trends keywords and DENV^a^ RT-PCR^b^ positive rate.

Keywords		Correlation (95% CI)	*P* value	Max cross-correlation^c^	Time lag^d^
**Dengue**					
	Keywords “dengue” + “dingue” and region “Martinique”	0.632 (0.531-0.714)	*<.001* ^e^	0.632	0 months
	Keywords “dengue” + “Martinique”	0.592 (0.484-0.681)	*<.001*	*0.643* ^f^	−*1 month*
	Topic “dengue” and region “Martinique”	0.675 (0.583-0.749)	*<.001*	0.675	0 months
**Dengue symptoms**					
	Keyword “symptome dengue” and region “Martinique”	0.436 (0.306-0.55)	*<.001*	*0.453*	−*1 month*
**Mosquito**					
	Keyword “mosquito” with various French spellings and region “Martinique”	0.217 (0.004-0.068)	*<.001*	0.217	0 weeks
**Aedes**					
	Keywords “aedes” and region “Martinique”	0.379 (0.243-0.501)	*<.001*	*0.394*	−*1 month*
	Topic “aedes” and region “Martinique”	0.242 (0.095-0.379)	*<.001*	*0.313*	−*2 months*

^a^DENV: dengue virus.

^b^RT-PCR: real-time reverse transcriptase polymerase chain reaction.

^c^Maximum cross-correlation.

^b^Time lag that results in the maximum cross-correlation.

^e^Italicized *P* values are significant.

^f^Italicized cross-correlations are statistically significant (details in Multimedia Appendices 2-5).

## Discussion

### Principal Findings

This study demonstrates the potential of real-world data for dengue outbreak monitoring. It indicates that multiple heterogeneous data sources, such as clinical data, vector data, and novel Big Data streams, should be leveraged simultaneously because they can all play a role in improving traditional dengue surveillance systems. Moreover, some data, such as the weekly hospitalization rates for thrombocytopenia, the weekly number of entomological interventions, and Google keywords, were not only significantly correlated with the weekly DENV RT-PCR positive rates, but their increase preceded the increase in RT-PCR positive results by 2 to 4 weeks.

An early response is crucial in dengue management because it can reduce mortality [18] and help stakeholders better anticipate needs and resources. In Martinique, the early signs identified in this study could be used to set up more hospital beds (including in the intensive care unit), increase staffing, particularly in emergency services and infectious diseases department, and increase the blood bank stock levels for patients with severe dengue who may need blood transfusions. Moreover, stakeholders could use them to justify requests for reinforcements from other territories (for Martinique, mostly from mainland France), medical equipment, and hospital staff. In addition, they could be used to notify earlier the Pan-American Health Organization, which is the Regional Office for the Americas of the World Health Organization [33], and help other islands to better prepare for an incoming outbreak.

Previous studies have already investigated the role of entomological data [34], inpatient data [35], and internet data streams [36] in dengue management, but few have assessed all these data sources simultaneously. In this study, we found that they should all be considered together rather than individually. Vector-based data tend to be underused [37], despite their central place in dengue surveillance, although we observed a rather strong correlation between the number of weekly entomological interventions and the increase in DENV RT-PCR positive rates. Therefore, they should be better integrated into the dengue surveillance system to improve its efficiency because both clinical surveillance and vector-based surveillance are essential for optimal dengue management [38]. The role of internet search engines in dengue surveillance has been frequently addressed in recent years [23,39]. Most studies were carried out in Asia and in larger American countries, such as Mexico and Brazil [24,25], and used a different approach based on weekly extracted data, which was not possible in our case. However, we found that even in Martinique, a smaller territory with a smaller population and, thus, with a lower data volume from internet data streams, Google queries were still correlated with an increase in the DENV RT-PCR positive rate. This means that they can also be used as part of surveillance systems across the islands of the Lesser Antilles. However, the methodological framework [29] still needs to be adapted to the size of these territories, and the simplest keywords and Google topics, when available, should be preferred over multiple spelling variations. With these small adaptations, we propose a way to offset the limitations related to smaller territories to use internet data streams in this context because their interest in emerging disease surveillance has been demonstrated in previous studies [40,41]. Overall, for smaller territories, the challenge lies in the small population size that leads to a lower weekly signal variability, thus complicating covariance estimation (and consequently the use of correlation methods). As most studies evaluating real data sources for dengue surveillance were based on correlation methods [22,37,42], we needed to confirm that these approaches were still applicable using a smaller sample. Despite these limitations, we managed to identify relevant indicators from all data sources to improve monitoring.

Moreover, most studies on real-world data sources used symptomatic cases as gold standard [37]. However, in practice, public health authorities do not rely solely on symptomatic cases for decision-making during an outbreak. Here, we compared our data sources to the actual gold standard used by stakeholders for decision-making, which is based on objective and subjective parameters and found a reliable objective proxy (ie, the weekly DENV RT-PCR positive rates) to assess our variables. Finally, dengue hospitalizations and the symptoms associated with severe dengue cases (thrombocytopenia and liver dysfunction symptoms) should be closely monitored in inpatients, especially in children, because they tend to precede the DENV RT-PCR positive rate increase by several weeks.

Our study also highlighted homogenous time lags across different data sources, despite their heterogeneity. This further demonstrates the importance of considering them globally rather than individually, although some of these correlations were low or moderate. For instance, an increase in hospitalized patients with liver dysfunction symptoms could prompt physicians to pay closer attention to the dengue hospitalization rate because both precede the increase in DENV RT-PCR by 5 weeks and 3 weeks, respectively. The capacity to identify variables that precede the DENV RT-PCR positive rate increase is very relevant for dengue management because a rapid and early response can influence outbreak severity [18].

### Limitations

Our method is promising but has some limitations. First, some correlations were very low, although they were statistically significant. Second, we did not include climate data because insufficient data were available for our time frame. Several studies have demonstrated the role of climate data (especially temperature, humidity, and rainfall) in dengue surveillance, but they were mostly carried out in Asian countries [43,44] and South America [45,46]. Few studies in the Caribbean region showed the role of rainfall and temperature in increasing the risk of dengue outbreaks. However, their time lags (between 7 weeks and 5 months) [47,48] were longer than the time lags we found for the other data sources. Nevertheless, this data source could have been relevant.

Third, our laboratory data did not include private sector biology laboratories, because they did not use RT-PCR techniques before the COVID-19 pandemic in 2020. Before this date, dengue diagnosis in private sector laboratories was based on NS1 antigen detection and needed sometimes to be confirmed by the more sensitive RT-PCR test at the hospital laboratory. It should be noted that the weekly number of DENV RT-PCR tests increased over time. Therefore, we used the weekly positive rate and not the weekly number of RT-PCR tests. Similarly, the World Health Organization dengue case classification and guidelines for hospitalization changed during the study period [49], and this may have influenced the results. Nevertheless, the rate of hospitalized patients with a dengue diagnosis was more strongly correlated with the DENV RT-PCR positive rate in our study.

Finally, concerning the entomological data, we only studied the correlation between the weekly number of interventions and the increase in the DENV RT-PCR positive rate, but we did not consider the number of mosquito clusters (ie, several clusters can be detected during 1 intervention). We focused on the simplest variable because vector control programs vary among the countries in this region [4,6], and we wanted to develop a common approach for all Caribbean territories. Furthermore, because entomological interventions tend to increase during outbreaks, we cannot rule out the influence of these practices on our results. Nevertheless, we could show that entomological interventions precede the increase in the DENV RT-PCR positive rate by 2 weeks.

Our approach does not intend to replace traditional monitoring systems based on syndromic surveillance, but to reduce the delays in these systems by leveraging data that are already routinely collected. These new data sources are readily available and can be easily implemented in the existing surveillance systems with minimal cost and training. However, their ability to predict future dengue outbreaks need to be thoroughly assessed, especially in smaller territories in the Lesser Antilles.

### Conclusions

Our study shows that real-world data are valuable data sources for dengue surveillance in Martinique. Several heterogeneous data sources are relevant, from clinical data to vector control data and Google Trends data. Their increase precedes the increase in dengue cases by several weeks, and therefore, they can help to improve traditional surveillance systems to provide an early response to dengue outbreaks. By improving the integration of many different sources, we might better respond to dengue outbreaks in endemic regions, as well as to other types of vector-borne diseases such as Zika and chikungunya.

## Appendix

Multimedia Appendix 1 International Classification of Diseases, 10th edition, codes of the selected diagnoses for inpatient data.

Multimedia Appendix 2 Cross-correlations between administrative data and dengue virus real-time reverse transcription polymerase chain reaction positive rate.

Multimedia Appendix 3 Cross-correlations between dengue diagnoses inpatients and dengue virus real-time reverse transcription polymerase chain reaction positive rate.

Multimedia Appendix 4 Cross-correlations between general dengue symptoms among inpatients and dengue virus real-time reverse transcription polymerase chain reaction positive rate.

Multimedia Appendix 5 Cross-correlations between specific dengue symptoms among inpatients and dengue virus real-time reverse transcription polymerase chain reaction positive rate.

Multimedia Appendix 6 Weekly correlations between Google Trends keywords and dengue virus real-time reverse transcription polymerase chain reaction positive rate.

Multimedia Appendix 7 Monthly correlations between Google Trends keywords and dengue virus real-time reverse transcription polymerase chain reaction positive rate.

Multimedia Appendix 8 Weekly cross-correlations between nonhospital data and dengue virus real-time reverse transcription polymerase chain reaction positive rate.

Multimedia Appendix 9 Monthly cross-correlations between Google Trends keywords and dengue virus real-time reverse transcription polymerase chain reaction positive rate.

## Abbreviations

AUC: area under the curve
CEDRE: Centre de Démoustication et de Recherche en Entomologie
DENV: dengue virus
ICD-10: International Classification of Diseases, 10th edition
PSAGE: Programme de surveillance, d’alerte et de gestion des épidémies de dengue
RSV: relative search volumes
RT-PCR: real-time reverse transcriptase polymerase chain reaction
