# Supplementary material for: The Role of Heterogenous Real-world Data for Dengue Surveillance in Martinique: Observational Retrospective Study
Source: JMIR Public Health Surveill. 2022 Dec 22;8(12):e37122. doi: 10.2196/37122 (PMC9816958; doi:10.2196/37122)
Supplement: Multimedia Appendix 1 [file publichealth_v8i12e37122_app1.docx]

**Multimedia Appendix 1. ICD-10^a^ codes of the selected diagnoses for inpatient data.**

| **ICD-10 Code** | **ICD-10 Title** |
| --- | --- |
| **Dengue** |  |
| A90^b^ | Dengue fever [classical dengue] |
| A91 ^b^ | Dengue haemorrhagic fever |
| A97.0 | Dengue without warning signs |
| A97.2 | Dengue with warning signs |
| A97.9 | Severe Dengue |
|  |  |
| **Coding errors** |  |
| A99 | Unspecified viral haemorrhagic fever |
| R50.9 | Fever, unspecified |
|  |  |
| **Severity symptoms** |  |
| E86 | Volume depletion |
| R57.0 | Cardiogenic shock |
| R57.1 | Hypovolaemic shock |
| R57.2 | Septic shock |
| R57.8 | Other shock |
| R57.9 | Shock, unspecified |
| R58 | Haemorrhage, not elsewhere classified |
|  |  |
| **Thrombocytopenia** |  |
| D69.4 | Other primary thrombocytopenia |
| D69.5 | Secondary thrombocytopenia |
| D69.6 | Thrombocytopenia, unspecified |
|  |  |
| **Hepatic symptoms** |  |
| B17.8 | Other specified acute viral hepatitis |
| B17.9 | Acute viral hepatitis, unspecified |
| B19.0 | Unspecified viral hepatitis with hepatic coma |
| B19.9 | Unspecified viral hepatitis without hepatic coma |
| K72.0 | Acute and subacute hepatic failure |
| K72.9 | Hepatic failure, unspecified |
| K75.9 | Inflammatory liver disease, unspecified |
| K77.0 | Liver disorders in infectious and parasitic diseases classified elsewhere |
| R16.0 | Hepatomegaly, not elsewhere classified |
| R16.1 | Splenomegaly, not elsewhere classified |
| R16.2 | Hepatomegaly with splenomegaly, not elsewhere classified |
| R74.0 | Elevation of levels of transaminase and lactic acid dehydrogenase [LDH] |
|  |  |
| **Neurologic symptoms** |  |
| G05.1 | Encephalitis, myelitis and encephalomyelitis in viral diseases classified elsewhere |
| G93.4 | Encephalopathy, unspecified |
| G94.3 | Encephalopathy in diseases classified elsewhere |

^a^ International Classification of Diseases, 10th Revision; ^b^ Former ICD-10 codes for dengue diagnosis
