# Supplementary figures and images for: The Role of Heterogenous Real-world Data for Dengue Surveillance in Martinique: Observational Retrospective Study
Source: JMIR Public Health Surveill. 2022 Dec 22;8(12):e37122. doi: 10.2196/37122 (PMC9816958; doi:10.2196/37122)

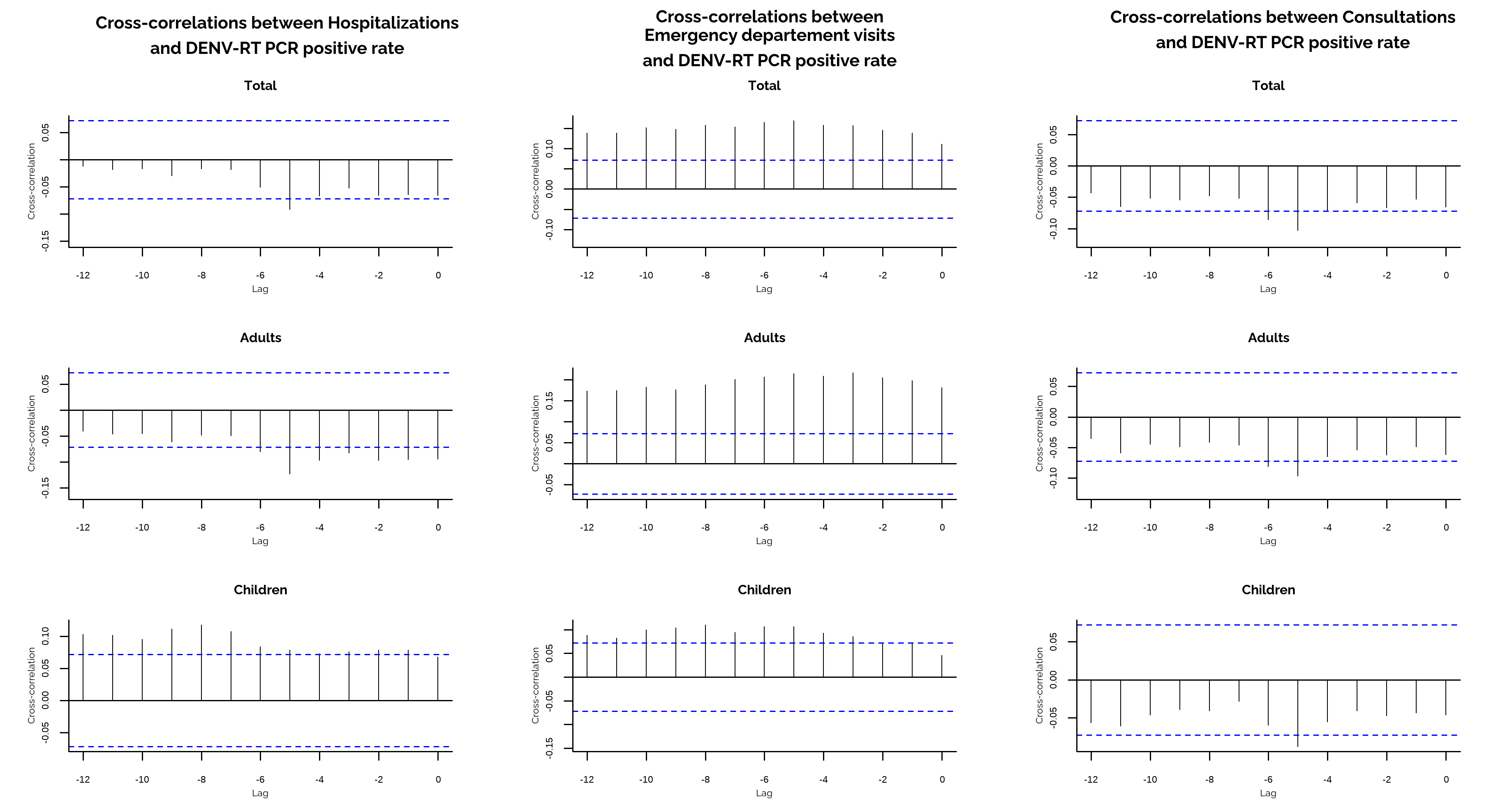

Supplement: Multimedia Appendix 2 [file publichealth_v8i12e37122_app2.png]

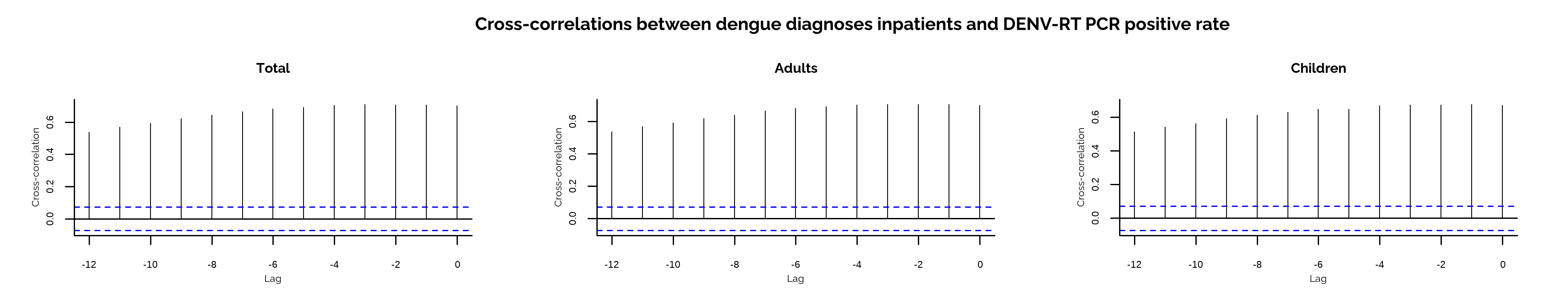

Supplement: Multimedia Appendix 3 [file publichealth_v8i12e37122_app3.png]

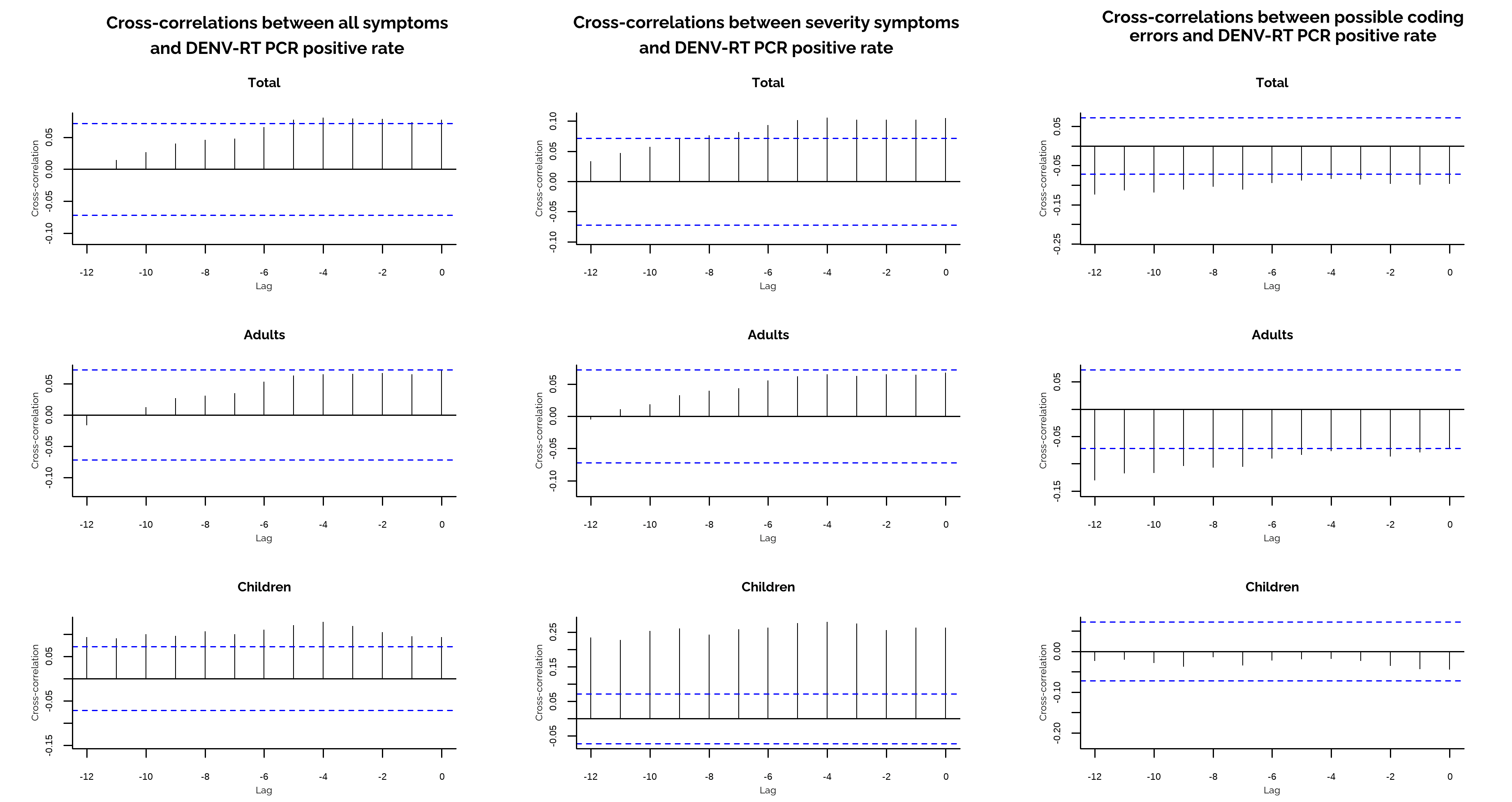

Supplement: Multimedia Appendix 4 [file publichealth_v8i12e37122_app4.png]

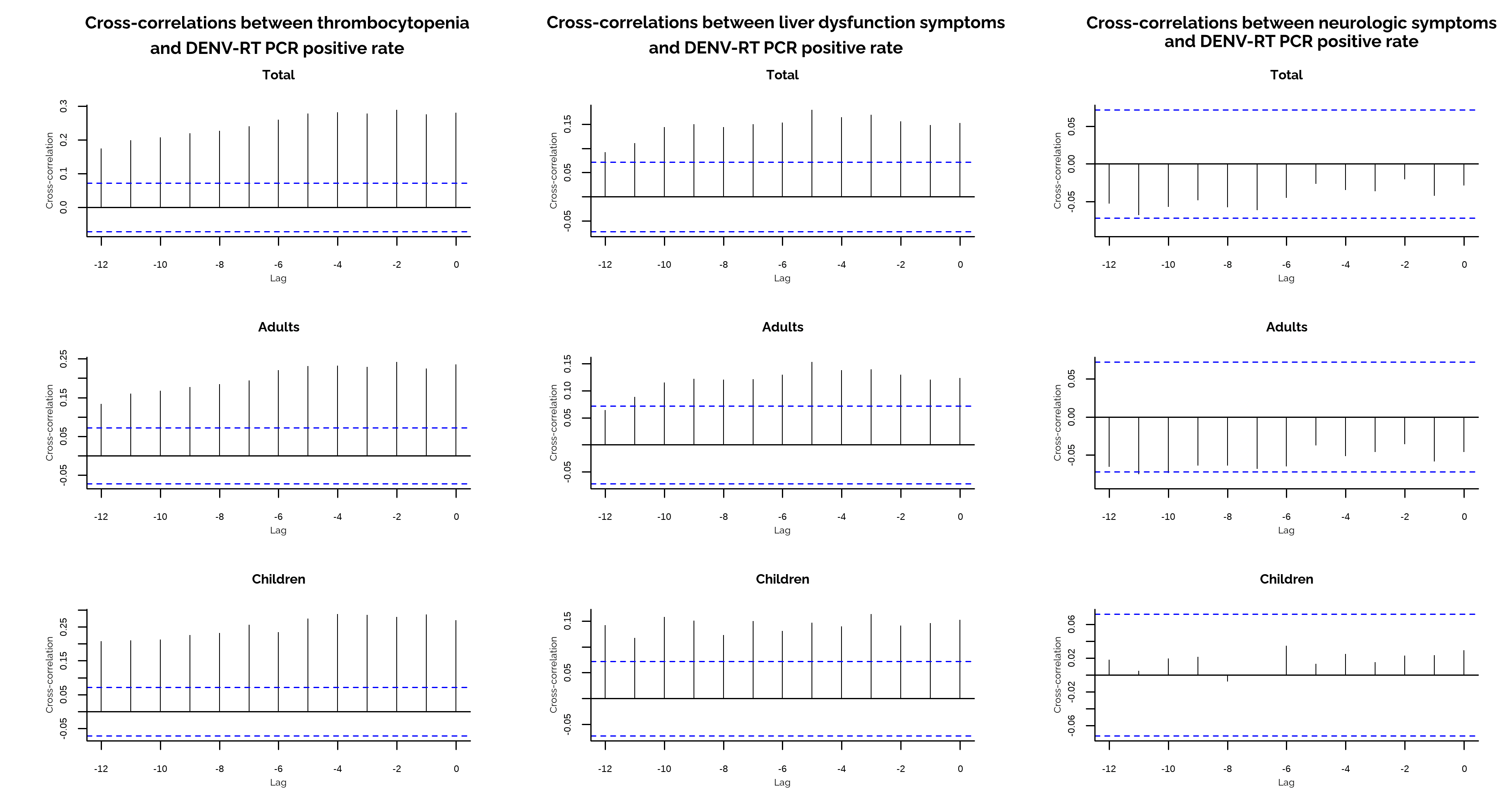

Supplement: Multimedia Appendix 5 [file publichealth_v8i12e37122_app5.png]

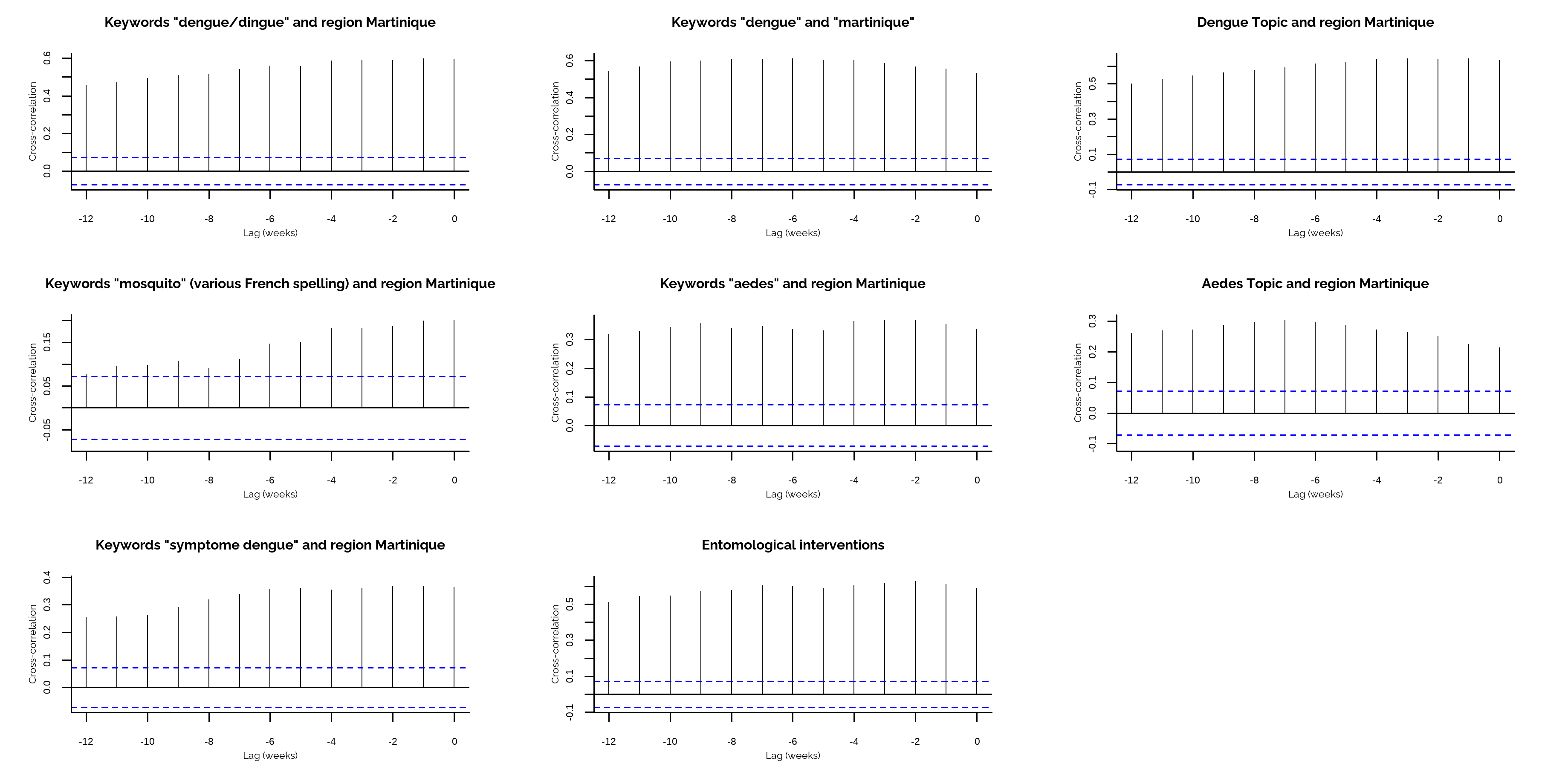

Supplement: Multimedia Appendix 8 [file publichealth_v8i12e37122_app8.png]

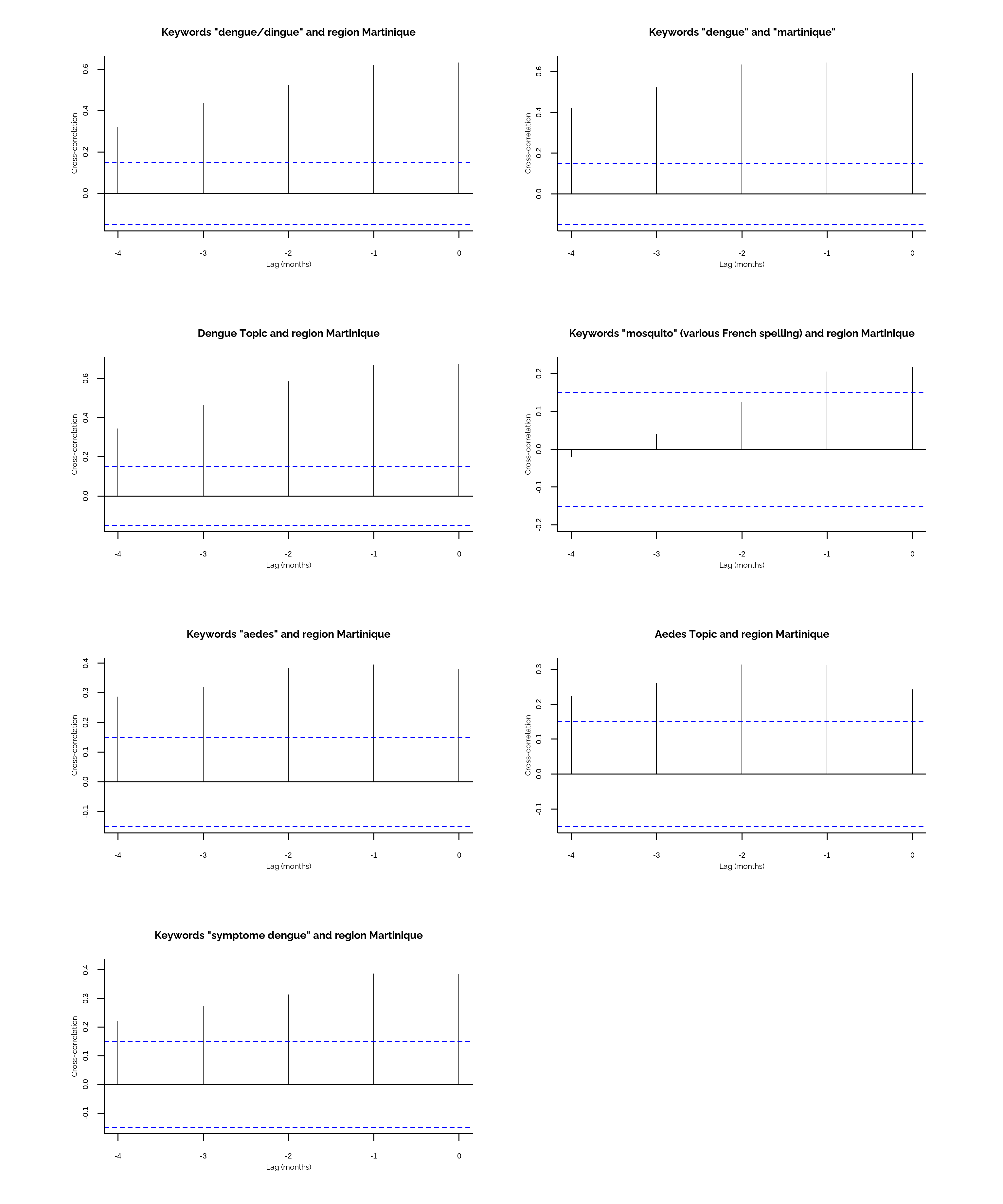

Supplement: Multimedia Appendix 9 [file publichealth_v8i12e37122_app9.png]
