# Supplementary material for: The Role of Heterogenous Real-world Data for Dengue Surveillance in Martinique: Observational Retrospective Study
Source: JMIR Public Health Surveill. 2022 Dec 22;8(12):e37122. doi: 10.2196/37122 (PMC9816958; doi:10.2196/37122)
Supplement: Multimedia Appendix 6 [file publichealth_v8i12e37122_app6.docx]

**Multimedia Appendix 6. Correlations between Google Trends keywords and DENV RT-PCR positive rate**

| **Keywords** | **Correlation** | **P-value** | **Confidence interval** | **Max cross-correlation ^a^** | | **Lag ^b^** |
| --- | --- | --- | --- | --- | --- | --- |
|  |  |  |  |  |  | |
| **Dengue** |  |  |  |  |  | |
| Keywords “dengue + dingue” and region: Martinique | 0.597 | **6.75 x 10^-73^** | [0.548;0.641] | 0.598 | - 1 week | |
| Keywords “dengue + dingue” and “martinique” | NA ^c^ | NA ^c^ | NA ^c^ | NA ^c^ | NA ^c^ | |
| Keywords “dengue” and “martinique” | 0.534 | **5.21 x 10^-56^** | [0.480;0.583] | 0.611 | -6 weeks | |
| Dengue Topic and region Martinique | 0.637 | **1.37 x 10^-85^** | [0.591;0.677] | 0.643 | - 3 weeks | |
| Keyword “dengue” | -0.016 | 0.654 | [-0.088;0.055] | -0.046 | - 7 weeks | |
|  |  |  |  |  |  | |
| **Dengue symptoms** |  |  |  |  |  | |
| Keyword “symptome dengue” and region Martinique | 0.412 | **6.65 x 10^-32^** | [0.351;0.47] | 0.435 | -3 weeks | |
| Keyword “symptome dengue” with various French spellings and region Martinique | 0.238 | **4.61 x 10^-11^** | [0.169;0.305] | 0.249 | +1 week | |
| Keywords “symptome dengue” with various French spellings and “martinique” | NA ^c^ | NA ^c^ | NA ^c^ | NA ^c^ | NA ^c^ | |
| Keywords “symptome dengue” with various French spellings | 0.222 | **9.66 x 10^-10^** | [0.152;0.289] | 0.246 | -5 weeks | |
| Keyword “symptome dengue” with various spellings and languages, region Martinique | 0.209 | **8.24 x 10^-9^** | [0.140;0.277] | 0.209 | 0 weeks | |
| Keyword “symptomes dengue” and region Martinique | 0.381 | **4.03 x 10^-27^** | [0.318;0.44] | 0.435 | -3 weeks | |
| Keywords “symptome dengue” with various spellings and languages and “martinique” | NA ^c^ | NA ^c^ | NA ^c^ | NA ^c^ | NA ^c^ | |
| Keywords “symptome dengue” with various spellings and languages | 0.215 | **3.31 x 10^-9^** | [0.145;0.282] | 0.221 | -3 weeks | |
|  |  |  |  |  |  | |
| **Mosquito** |  |  |  |  |  | |
| Keyword mosquito and region: Martinique | -0.086 | **1.88 x 10^-2^** | [-0.157;-0.014] | -0.106 | -6 weeks | |
| Keyword mosquito | -0.049 | **1.79 x 10^-1^** | [-0.121;0.0223] | -0.101 | -10 weeks | |
| Keyword mosquito with various spellings and languages and region: Martinique | 0.028 | **4.48 x 10^-1^** | [-0.044;0.100] | 0.033 | +1 week | |
| Keyword mosquito with various French spellings and region: Martinique | 0.200 | **3.58 x 10^-8^** | [0.130;0.268] | 0.200 | 0 weeks | |
| Mosquito Topic and region: Martinique | 0.068 | **6.36 x 10^-2^** | [-0.004;0.139] | 0.098 | +8 weeks | |
| Keywords “mosquito” and “martinique” | -0.088 | **1.68 x 10^-2^** | [-0.158;-0.016] | -0.142 | +3 weeks | |
|  |  |  |  |  |  | |
| **Aedes aegypti** |  |  |  |  |  | |
| Keyword “aedes aegypti” and region Martinique | 0.109 | **3.06 x 10^-3^** | [0.037;0.179] | 0.112 | -1 week | |
| Keywords “aedes aegypti” and “martinique” | NA ^c^ | NA ^c^ | NA ^c^ | NA ^c^ | NA ^c^ | |
| Keyword “aedes aegypti” | -0.098 | **7.66 x 10^-3^** | [-0.169;-0.026] | -0.104 | -4 weeks | |
| Aedes aegypti Topic and region Martinique | 0.115 | **1.66 x 10^-3^** | [0.044;0.185] | 0.119 | -2 weeks | |
|  |  |  |  |  |  | |
| **Aedes** |  |  |  |  |  | |
| Keywords aedes and region Martinique | 0.339 | **1.99 x 10^-21^** | [0.273;0.401] | 0.369 | -3 weeks | |
| Keywords “aedes” and “martinique” | NA ^c^ | NA ^c^ | NA ^c^ | NA ^c^ | NA ^c^ | |
| Keyword “aedes” | -0.092 | **1.22 x 10^-2^** | [-0.163;-0.02] | -0.100 | -7 weeks | |
| Aedes Topic and region Martinique | 0.214 | **3.71 x 10^-9^** | [0.591;0.677] | 0.304 | -7 weeks | |

^a^ Maximum cross-correlation

^b^ Time lag that results in the maximum cross-correlation

^c^ Not enough data available
