# Supplementary material for: The Role of Heterogenous Real-world Data for Dengue Surveillance in Martinique: Observational Retrospective Study
Source: JMIR Public Health Surveill. 2022 Dec 22;8(12):e37122. doi: 10.2196/37122 (PMC9816958; doi:10.2196/37122)
Supplement: Multimedia Appendix 7 [file publichealth_v8i12e37122_app7.docx]

**Multimedia Appendix 7. Monthly correlations between Google Trends keywords and DENV RT-PCR positive rate**

| **Keywords** | **Correlation** | **P-value** | **Confidence interval** | **Max cross-correlation ^a^** | | **Lag ^b^** |
| --- | --- | --- | --- | --- | --- | --- |
|  |  |  |  |  |  | |
| **Dengue** |  |  |  |  |  | |
| Keywords “dengue + dingue” and region: Martinique | 0.632 | **2.62 x 10^-20^** | [0.531;0.714] | 0.632 | 0 months | |
| Keywords “dengue + dingue” and “martinique” | NA ^c^ | NA ^c^ | NA ^c^ | NA ^c^ | NA ^c^ | |
| Keywords “dengue” and “martinique” | 0.592 | **1.96 x 10^-17^** | [0.484;0.681] | 0.643 | **-1 month** | |
| Dengue Topic and region Martinique | 0.675 | **6.36 x 10^-24^** | [0.583;0.749] | 0.675 | 0 months | |
| Keyword “dengue” | -0.027 | 0.726 | [-0.177;0.124] | -0.048 | -2 months | |
|  |  |  |  |  |  | |
| **Dengue symptoms** |  |  |  |  |  | |
| Keyword “symptome dengue” and region Martinique | 0.436 | **2.79 x 10^-9^** | [0.306;0.55] | **0.453** | **-1 month** | |
| Keyword “symptome dengue” with various French spellings and region Martinique | 0.384 | **2.31 x 10^-7^** | [0.248;0.505] | -0.386 | -1 month | |
| Keywords “symptome dengue” with various French spellings and “martinique” | NA ^c^ | NA ^c^ | NA ^c^ | NA ^c^ | NA ^c^ | |
| Keywords “symptome dengue” with various French spellings | 0.237 | **1.84 x 10^-3^** | [0.090;0.374] | 0.245 | **-1 month** | |
| Keyword “symptome dengue” with various spellings and languages, region Martinique | 0.198 | **9.77 x 10^-3^** | [0.049;0.338] | 0.198 | 0 months | |
| Keyword “symptomes dengue” and region Martinique | 0.429 | 5.27 x 10^-9^ | [0.298;0.544] | 0.487 | -1 month | |
| Keywords “symptome dengue” with various spellings and languages and “martinique” | NA ^c^ | NA ^c^ | NA ^c^ | NA ^c^ | NA ^c^ | |
| Keywords “symptome dengue” with various spellings and languages | 0.234 | **2.17x 10^-3^** | [0.086;0.371] | 0.239 | -1 month | |
|  |  |  |  |  |  | |
| **Mosquito** |  |  |  |  |  | |
| Keyword mosquito and region: Martinique | -0.083 | **0.284** | [-0.230;-0.069] | -0.097 | -1 month | |
| Keyword mosquito | -0.056 | **0.472** | [-0.204;0.096] | -0.078 | -2 months | |
| Keyword mosquito with various spellings and languages and region: Martinique | 0.033 | **0.667** | [-0.012;0.183] | 0.037 | +1 month | |
| Keyword mosquito with various French spellings and region: Martinique | 0.217 | **3.58 x 10^-8^** | [0.004;0.068] | 0.217 | 0 months | |
| Mosquito Topic and region: Martinique | -0.056 | **0.47** | [-0.096;0.204] | 0.078 | -2 months | |
| Keywords “mosquito” and “martinique” | -0.092 | **0.232** | [-0.239;-0.059] | -0.095 | +1 month | |
|  |  |  |  |  |  | |
| **Aedes aegypti** |  |  |  |  |  | |
| Keyword “aedes aegypti” and region Martinique | 0.114 | **0.138** | [-0.037;0.260] | 0122 | -1 month | |
| Keywords “aedes aegypti” and “martinique” | NA ^c^ | NA ^c^ | NA ^c^ | NA ^c^ | NA ^c^ | |
| Keyword “aedes aegypti” | -0.108 | **0.160** | [-0.255;-0.043] | -0.118 | -2 months | |
| Aedes aegypti Topic and region Martinique | 0.126 | **0.101** | [-0.025;0.272] | 0.129 | -1 month | |
|  |  |  |  |  |  | |
| **Aedes** |  |  |  |  |  | |
| Keywords aedes and region Martinique | 0.379 | **3.37 x 10^-7^** | [0.243;0.501] | **0.394** | **-1 month** | |
| Keywords “aedes” and “martinique” | NA ^c^ | NA ^c^ | NA ^c^ | NA ^c^ | NA ^c^ | |
| Keyword “aedes” | -0.099 | **0.197** | [-0.163;-0.02] | -0.105 | **-2 months** | |
| Aedes Topic and region Martinique | 0.242 | **1.47 x 10^-3^** | [0.095;0.379] | **0.313** | **-2 months** | |

^a^ Maximum cross-correlation

^b^ Time lag that results in the maximum cross-correlation

^c^ Not enough data available
